# Individual-level socioeconomic status and contact or familiarity with people with mental illness: a cross-sectional study in Wuhou District, Chengdu, Southwest China

**DOI:** 10.1186/s12875-021-01422-y

**Published:** 2021-04-09

**Authors:** Mengmeng Wang, Ya Wang, Jiajun Xu, Na Meng, Xiaolin Li, Zheng Liu, Junqiang Huang

**Affiliations:** 1 West China School of Nursing, Sichuan University/Department of Nursing, West China Hospital, Sichuan University, Chengdu, 610041 China; 2 Department of Nursing, West China Hospital, Sichuan University/West China School of Nursing, Sichuan University, Chengdu, 610041 China; 3 Mental Health Center, West China Hospital, Sichuan University, Guoxuexiang No.37, Chengdu, 610041 Sichuan China; 4 Mental Health Center, West China Hospital, Sichuan University/West China School of Nursing, Sichuan University, Chengdu, 610041 China; 5 Chengdu Dekang Hospital, Sichuan University, Chengdu, 610041 China

**Keywords:** Attitudes, Mental illness, Public, Social distance, Stigma

## Abstract

**Background:**

People with mental illness (PWMI) often suffer from public stigma, which can make them unwilling to seek help and reduce access to early treatment. The aims of this study were to determine attitudes towards PWMI among the general public in a Chinese sample and to explore the relationships with sociodemographic characteristics.

**Methods:**

A community-based, cross-sectional study was conducted from March to June 2019. The participants’ attitudes towards PWMI were evaluated by the Chinese version of the Social Distance Scale (SDSC). An independent-sample T-test and one-way ANOVA were used to determine the association of categorical variables with the outcome variable. Multiple linear regression and Spearman correlations were computed to explore the correlation between SDSC scores and individual-level socioeconomic status (SES).

**Results:**

A total of 1437 participants were recruited, and their total SDSC score was 12.53 (SD: 3.11). Univariate analysis results showed that age, education level, educational attainment, and individual-level SES as well as whether they were caregivers/family members of PWMI were correlated with SDSC scores. The results of regression analysis showed a significant effect caused by contact or familiarity with PWMI (B = -1.134, β = -.190, *P* < 0.001), as well as for individual-level SES (B = -.339, β = -.110, *P* < 0.001). Spearman correlation results showed that SDSC scores were negatively correlated with individual-level SES (*r* = -.078, *p* < 0.01) and contact or familiarity with PWMI (*r* = -.168, *p* < 0.001).

**Conclusion:**

This study reveals that public stigma towards PWMI is common in Southwest China. Individuals who are not a family member or a caregiver of PWMI or have low education level or low individual-level SES need to be provided more anti-stigma interventions. Contacting with PWMI is also a potentially beneficial measure to reduce social distance.

**Supplementary Information:**

The online version contains supplementary material available at 10.1186/s12875-021-01422-y.

## Background

Public stigma towards people with mental illness (PWMI) is harsh and widespread throughout the world [1–3], and this stigma is a significant barrier to recovery for PWMI. Public stigma may affect the willingness to seek help [4, 5], reduce access to early treatment [6], and have a negative impact on work, personal relationships and even marriage [7, 8].

Additionally, public stigma can lead to further harm when internalized, which occurs when individuals become aware of public stigma, agree with it and apply it to themselves [9, 10]. A high level of internalized stigma is associated with low levels of informal help-seeking behaviours and high suicide rates [11]. Moreover, public stigma affects not only individuals but also entire families, as the family members of PWMI may experience discrimination and stigma [12–14], which are associated with their self-esteem and caregiver burden. Therefore, it is very important to focus on public attitudes towards PWMI.

A growing body of literature has reported an association between stigma and sociodemographic attributes such as gender, age, marital status, education level and income [15–17]; religious devotion [18, 19]; knowledge about mental illness [20, 21]; and contact, familiarity or personal acquaintance with PWMI [22–24]. A few studies have explored the relationship between individual-level socioeconomic status (SES) and stigma in the context of people with HIV or suicidal behaviour [25, 26]. However, the relationship between individual-level SES and mental illness stigma is unclear.

Moreover, although many studies have focused on factors that influence public attitudes towards PWMI, the results of studies across different cultures are inconsistent [27–29]. A review of studies on public attitudes towards PWMI found that only 15% of the reviewed studies (a total of 61 articles published between 1990 and 2004) were conducted in non-Western countries the [30]. A review study also showed that public stigma, stereotypes, and prejudices associated with violence, unpredictability, and disability in Latin America and the Caribbean were similar to those in many countries around the world [31]. Similarly, stigmatizing beliefs, actions and attitudes towards mental illness treatment are prevalent in Arab culture [32].

Furthermore, an overview of stigma towards PWMI in six Asian societies found that PWMI in Asian societies were regarded as less socially accepted and viewed as having personal weaknesses [33]. A study exploring stigma towards PWMI in Indonesia showed that income level was associated with stigma [15]. In Singapore, Subramaniam, M. et al. found that having lower education and lower income was significantly associated with a higher level of stigma [34]. In China, attitudes towards PWMI have received more attention in recent years; however, most studies have been performed in Hong Kong, Taiwan and first-tier cities (e.g., Beijing, Shanghai and Guangzhou) on the mainland [35]. Therefore, it is necessary to explore public attitudes towards PWMI in other parts of China.

According to previous studies, the factors that influence public attitudes towards PWMI differ by culture. Attitudes of the general public towards PWMI are more negative in Asian societies than in other societies. However, few studies have been conducted in non-first-tier Chinese cities, which could be different. Therefore, the aims of this study were to determine attitudes towards PWMI among the general public in Wuhou District, Chengdu, Southwest China, and to explore the relationships with sociodemographic characteristics. Our hypothesis is that people with higher individual-level SES show more positive attitudes towards PWMI. It is hoped that the study findings may help with anti-stigma campaigns aiming to reduce public stigma towards PWMI. Furthermore, this study could provide guidance for government policies.

## Methods

### Design and settings

A community-based, cross-sectional study was conducted in Wuhou District, Chengdu, Southwest China, from March to June 2019. Wuhou District, which is one of 9 districts in Chengdu city, has 13 sub-districts. The household registration of Wuhou District is 478,829, with a total population of 1,218,232 at the end of 2017.

### Participants and sample size

Multistage sampling was used in this study. Wuhou District was selected by a lottery among the 9 districts in Chengdu. Ten resident communities were selected by cluster random sampling from 13 sub-districts. Recruited by convenience sampling in the selected resident communities, participants were residents aged 18 or over in Wuhou District.

We used the formula $$(n = (Z_{{{\raise0.7ex\hbox{$\alpha $} \!\mathord{\left/ {\vphantom {\alpha 2}}\right.\kern-\nulldelimiterspace} \!\lower0.7ex\hbox{$2$}}}} )^{2} \times p\left( {1 - p} \right)/e^{2} )$$ for a single population proportion to compute the sample size. The margin of error was taken as 5%, with a 95% confidence interval. Because there are no publicly available data on specific areas of study, the total sample size was calculated based on the assumption of a 50% level of public stigma. Therefore, the estimated sample size was 385. After the design effect was considered, the calculated sample size increased to 770. We assumed a 20% nonresponse rate, so the minimum sample size was 924.

### Data collection

The resident health survey team was established and composed of healthcare workers of community healthcare service centres and volunteers. There were 10 investigation groups, with two investigators in each group who were trained to administer the questionnaires. The residents’ committee staff accompanied the investigators into the community to assist in the investigation. The questionnaires were administered face to face. Participants were informed about the aim of the study and invited to participate, and they filled out the questionnaire independently and anonymously. Informed consent was obtained from all subjects, and all methods were performed in accordance with the relevant guidelines and regulations.

### Measures

#### Demographic variables

The questionnaire collected data on individual demographic variables including age, gender, marital status, education attainment, place of birth, current residence, only child status and whether the respondent was a caregiver for an elderly person.

#### Contact or familiarity with PWMI

Data were collected from 3 dichotomous (yes or no) questions concerning contact or familiarity with PWMI (1: Are you a caregiver of PWMI? 2: Have you experienced mental illness? 3: Do you have a family member who has experienced mental illness?). If the respondent answered ‘no’ to all of the above questions, he or she was assigned to the ‘no contact or familiarity with PWMI’ category; if the answers were ‘yes’ to all questions, he or she was assigned to the ‘all contact or familiarity with PWMI’ category; and the others were assigned to the ‘contact or familiarity with PWMI’ category.

#### Individual-level SES

Education, occupation and income are usually used as indicators of SES. We used self-reported education brackets (1 = primary schools and below, 2 = junior high school, 3 = high school, 4 = technical secondary school and junior college, 5 = bachelor’s degree or above), economic status brackets or monthly household income (1 = very poor (1500–2999 RMB), 2 = relatively poor (3000–4999 RMB), 3 = general (5000–9999 RMB), 4 = relatively well off (10,000–14,999 RMB), and 5 = very well off (≥ 15,000 RMB)) and occupation to assess individual-level SES. The occupations were classified as 5 = large company managers, public institutions, or professional and technical personnel; 4 = small company managers or assistive technicians; 3 = ordinary staff or clerks; 2 = skilled workers; and 1 = farmers, temporary workers or unemployed individuals. The overall individual-level SES was the sum of education, occupation and income. The scores for individual-level SES were divided into 5 classes (low class = 3–5, medium–low class = 6–8, medium class = 9–11, medium–high class = 12–14, and high class = 15) [36, 37] in the context of mainland China. In this study, the medium–high class and high class were grouped together.

#### Social Distance Scale-Chinese version (SDSC)

The Social Distance Scale (SDS) was created by Whatley to assess discrimination against mental illness [38]. A Chinese version of the SDS was created and verified, with a Cronbach’s alpha value of 0.733 [39] (the SDSC can be obtained at https://onlinelibrary.wiley.com/doi/full/10.1111/j.1440-1819.2009.01922.x.). The SDSC is an 8-item self-report inventory that assesses social distance by asking about private and social relationships. Example items include ‘It is best not to associate with colleagues who have been treated for mental illness’ and ‘It is wrong to shy away from people with mental illness’. Responses are given on a 4-point Likert (0–3 points) scale ranging from ‘strongly disagree’ to ‘strongly agree’, with higher scores indicating more negative attitudes. Items 2 and 7 are reverse scored, and the total score is calculated as the sum of items.

### Statistical analysis

The program SPSS v. 26.0 was used for the statistical analysis. Before starting the analysis, we performed reverse coding for negative statements. Descriptive statistics (frequencies and percentages) were used for the sociodemographic variables. Means (standard deviations) were used for the quantitative variables if they had a normal distribution. An independent-sample T-test or one-way ANOVA was used to determine the association of the categorical variables with the outcome variable. Finally, multiple linear regression was computed to explore the factors affecting general public attitudes towards PWMI. The table listing the independent variables is shown in Additional file 1. Spearman correlations were computed to explore the correlation among SDSC, individual-level SES, and contact or familiarity with PWMI. Interactions between individual-level SES and contact or familiarity with PWMI were considered. *P* < 0.05 indicated significance in all tests.

## Results

Of the 1600 participants approached for this study, 163 participants refused to participate, and a total of 1437 participants were recruited. The participants had a mean age of 48.28 years (SD: 16.35), and most were female (58.4%) and married or living with a partner (75.6%). The sociodemographic characteristics of the participants are shown in Table 1.

**Table 1:** Differences in SDSC of different sociodemographic characteristics (Means ± Standard Deviations) (*N* = 1437)

Variable	N (%)	SDS	t/F	*P*
Gender				
Male	598 (41.6)	12.54 ± 3.15	0.159	0.874
Female	839 (58.4)	12.52 ± 3.07		
Age group (year)				
18–30	251 (17.5)	12.49 ± 3.02	5.318^a^	< 0.001
31–45	413 (28.7)	12.12 ± 3.17		
46–60	379 (26.4)	12.42 ± 3.08		
61–75	326 (22.7)	13.12 ± 3.06		
More than 75	68 (4.7)	13.01 ± 3.01		
Marital status				
Unmarried	229 (15.9)	12.26 ± 3.30	2.773	0.063
Married or live together	1087 (75.6)	12.53 ± 3.06		
Divorced or widowed	121 (8.4)	13.08 ± 3.08		
Education level				
Primary schools and below	238 (16.6)	12.90 ± 3.38	3.915^b^	0.004
Junior high school	343 (23.9)	12.69 ± 3.17		
High school	357 (24.8)	12.70 ± 2.93		
Technical secondary school and junior college	237 (16.5)	11.94 ± 2.87		
Bachelor’s degree or above	262 (18.2)	12.30 ± 3.13		
Educational attainment (year)				
Less than or equal to 9	572 (39.8)	12.83 ± 3.30	2.875	0.004
More than 9	865 (60.2)	12.34 ± 2.95		
Occupation				
Large company managers, public institutions, or professional and technical personnel	560 (39.0)	12.68 ± 3.29	2.357	0.052
Small company managers or assistive technicians	98 (6.8)	13.19 ± 2.82		
Ordinary staff or clerks	318 (22.1)	12.28 ± 2.92		
Skilled workers	61 (4.2)	12.08 ± 3.01		
Farmers, temporary workers or unemployed individuals	400 (27.8)	12.44 ± 3.05		
Economic status/Monthly household income				
Very poor (1500–2999 RMB)	83 (5.8)	12.64 ± 2.62	1.304	0.266
Relatively poor (3000–4999 RMB)	167 (11.6)	12.23 ± 3.38		
General (5000–9999 RMB)	1024 (71.3)	12.63 ± 3.11		
Relatively well-off (10,000–14,999 RMB)	154 (10.7)	12.14 ± 3.03		
Very well off (≥ 15,000 RMB)	9 (0.6)	12.67 ± 2.65		
Socioeconomic status				
Low class	236 (16.4)	12.94 ± 3.48	2.956^c^	0.031
Medium–low class	512 (35.6)	12.66 ± 3.04		
Medium class	367 (25.5)	12.34 ± 2.91		
Medium–high class and High class	322 (22.4)	12.25 ± 3.12		
Place of birth				
Urban	713 (49.6)	12.53 ± 3.22	-0.069	0.945
Town or rural area	724 (50.4)	12.54 ± 2.99		
Current residence				
Urban	1313 (91.4)	12.52 ± 3.16	-0.780	0.437
Town or rural area	124 (8.6)	12.70 ± 2.45		
Are you the only child in the family?				
Yes	476 (33.1)	12.41 ± 3.31	-1.025	0.306
No	961 (66.9)	12.59 ± 3.00		
Are you a caregiver for an elderly person?				
Yes	415 (28.9)	12.56 ± 3.25	0.183	0.855
No	1022 (71.1)	12.52 ± 3.05		
Are you a caregiver of PWMI?				
Yes	465 (32.4)	11.94 ± 2.89	-5.174	< 0.001
No	972 (67.6)	12.81 ± 3.17		
Have you experienced a mental illness?				
Yes	198 (13.8)	12.18 ± 3.64	-1.494	0.136
No	1239 (86.2)	12.59 ± 3.01		
Do you have a family member who has experienced mental illness?				
Yes	277 (19.3)	12.14 ± 3.27	-2.321	0.020
No	1160 (80.7)	12.63 ± 3.06		
Contact or familiarity with PWMI group				
No-contact or familiarity with PWMI at all	759 (52.8)	13.00 ± 3.04	18.616^d^	< 0.001
Contact or familiarity with PWMI	663 (46.1)	12.03 ± 3.09		
All-contact or familiarity with PWMI	15 (1.0)	11.40 ± 3.46		

Data were compared by independent sample T test or one-way ANOVA

*PWMI*  People with mental illness

^a^There were significant post hoc (LSD) tests between age (18–30 years) and age (61–75 years) (*p* = 0.015), between age (31–45 years) and age (61–75 years) (*p* < .001), between age (31–45 years) and age (> 75 years) (*p* = 0.028) and between age (46–60 years) and age (61–75 years) (*p* = 0.003), showing that the age group (> 60 years) has highest SDSC scores

^b^There were significant post hoc (LSD) tests between educational level (primary schools and below) and educational level (technical secondary school and junior college) (*p* = 0.001), between educational level (primary schools and below) and educational level (bachelor’s degree or above) (*p* = 0.030), between educational level (junior high school) and educational level (technical secondary school and junior college) (*p* = 0.004) and between educational level (high school) and educational level (technical secondary school and junior college) (*p* = 0.003), showing that the educational level group (technical secondary school and junior college) has lowest SDSC scores

^c^There were significant post hoc (LSD) tests between socioeconomic status (low class) and socioeconomic status (medium class) (*p* = 0.021) and between socioeconomic status (low class) and socioeconomic status (medium–high class and high class) (*p* = 0.010), showing that the educational level group (medium–high class and high class) has lowest SDSC scores

^d^There were significant post hoc (LSD) tests between contact or familiarity with PWMI group (no-contact or familiarity with PWMI) and contact or familiarity with PWMI group (contact or familiarity with PWMI) (*p* < 0.001), between contact or familiarity with PWMI group (no-contact or familiarity with PWMI) and contact or familiarity with PWMI group (all-contact or familiarity with PWMI) (*p* = 0.046), showing that the contact or familiarity with PWMI group (no-contact or familiarity with PWMI) has highest SDSC scores

Table 1 shows that some sociodemographic characteristics of the general public are significant. Age, education level, educational attainment, contact or familiarity with PWMI, and individual-level SES were extracted as significant factors associated with the general public attitudes towards PWMI. In addition, people who were caregivers or family members of PWMI exhibited less social distance from PWMI.

Table 2 presents the social distance from PWMI among the general public. The total SDSC score was 12.53 (SD: 3.11), the work relations score was 4.32 (SD: 1.59), the shallow relationships score was 3.61 (SD: 1.24), and the employment score was 1.54 (SD: 0.73).

**Table 2:** Social distance toward PWMI among the general public (*n* = 1437)

Variables	SDSC (Mean ± SD)
The total score of Social Distance Scale	12.53 ± 3.11
Work relations^a^	4.32 ± 1.59
Shallow relationships^b^	3.61 ± 1.24
Employment^c^	1.54 ± 1.54
Eight questions	
1. It is best not to associate with colleagues who have been treated for mental illness	1.11 ± 0.70
2^d^. It is wrong to shy away from people with mental illness	1.46 ± 0.68
3. It would bother me to work with colleagues who had been in a mental hospital	1.81 ± 0.75
4. I would be against any daughter of mine marrying a man who had been in a mental hospital	1.54 ± 0.73
5. I would rather not hire a person with mental illness who had been in a mental hospital	1.75 ± 0.79
6. Leaders with psychosis who have been in a mental hospital should not be allowed to lead	1.79 ± 0.76
7^d^. If I needed a babysitter, I would be willing to hire a woman with a history of mental illness	1.19 ± 0.66
8. I would not ride in a taxi driven by someone who had been in a mental hospital	1.88 ± 0.859

*SDSC* Social Distance Scale-Chinese version, *PWMI* People with mental illness

^a^The work relations subscale includes item 1, item 3 and item 6

^b^The shallow relationships include item 4 and item 8

^c^The employment subscale includes item 5

^d^Item 2 and item 7 were reversal items and were reverse scores

The results of regression analysis (Table 3) revealed a significant effect caused by contact or familiarity with PWMI (B = -1.134, β = -0.190, *P* < 0.001), with the group with all contact or familiarity with PWMI showing less social distance. A significant effect was also found for individual-level SES (B = -0.339, β = -0.110, *P* < 0.001), where a higher individual-level SES was associated with less social distance.

**Table 3:** Multiple linear regression analysis with SDS as dependent variable (*N* = 1437)

	B	SE	Beta	t	*P*	95% CI	
Constant	15.073	.464		32.515	< .001	14.164	15.983
Age group							
18–30 years	Reference						
31–45 years	-.279	.264	-.041	-1.058	.290	-.797	.239
46–60 years	-.063	.287	-.009	-.221	.825	-.626	.499
61–75 years	.372	.301	.050	1.237	.216	-.218	.962
More than 75 years	.138	.446	.009	.308	.758	-.738	1.013
Marital status							
Unmarried	Reference						
Married or live together	-.042	.253	-.006	-.165	.869	-.537	.454
Divorced or widowed	.466	.374	.042	1.244	.214	-.269	1.200
Contact or familiarity with PWMI group	-1.134	.167	-.190	-6.807	< .001	-1.461	-.808
Socioeconomic status	-.339	.089	-.110	-3.793	< .001	-.514	-.164

*R*^2^ = 0.050, *R*^2^ adj. = 0.045

The Spearman correlation results (Table 4) suggested that SDSC scores were negatively correlated with individual-level SES (r = -0.078, *p* < 0.01) and contact or familiarity with PWMI (r = -0.168, *p* < 0.001).

**Table 4:** Correlation coefficients between SDSC and SES among the general public (*n* = 1437)

Spearman’s rho correlation	1	2	3
SDSC			
1. Total SDSC	1		
2. SES	-.078**	1	
3. Contact or familiarity with PWMI group	-.168***	-.290***	1

*SDSC* Social Distance Scale-Chinese version, *SES* Socioeconomic status, *PWMI* People with mental illness

^**^p < 0.01, *** *p* < 0.001

**Fig. 1:**
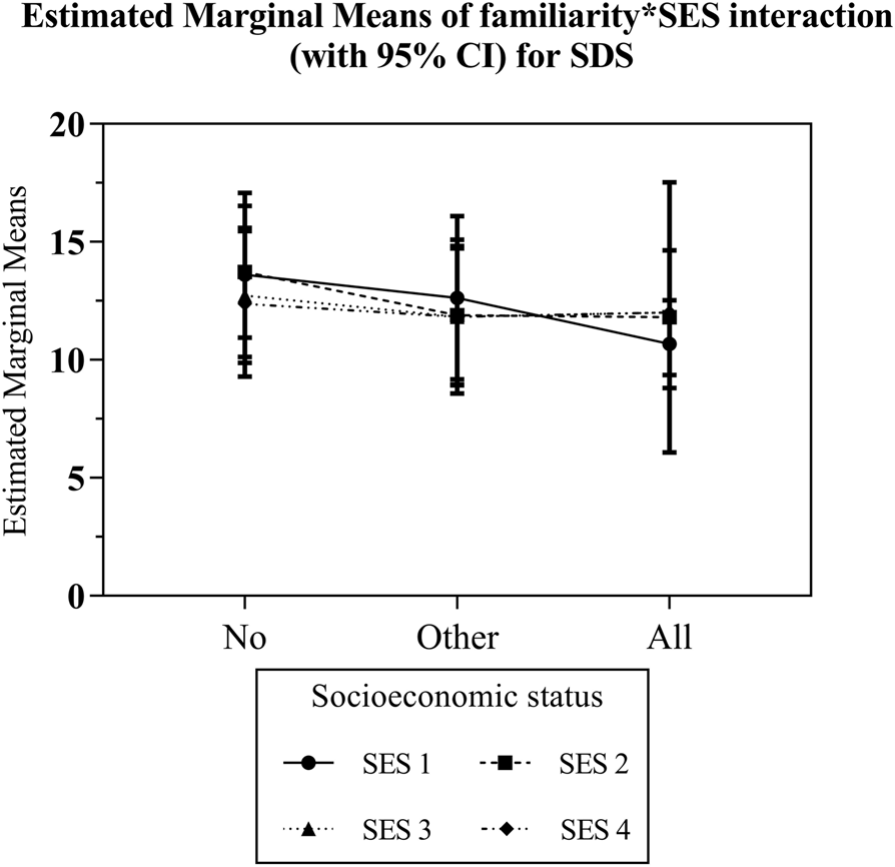
Estimated marginal means with 95% confidence intervals for the interaction between the level of contact or familiarity with PWMI and SES for SDS

Figure 1 shows the estimated margins from the regression analysis on public attitudes for each category of familiarity and individual-level SES within the interaction term. The tests of between-subjects effects did not yield a statistically significant result (*p* = 0.103).

## Discussion

This study assessed attitudes towards PWMI among the general public and determined the associations between attitudes and sociodemographic characteristics. Although this kind of study has been carried out elsewhere in the world, to the best of our knowledge, this study is the first to investigate the general public in Southwest China and to determine the relationship between individual-level SES and public attitudes. Our sample does not represent the entire population of the general public across the country, but this study still provides reliable data on attitudes towards PWMI in Southwest China.

### Factors associated with attitudes towards PWMI

The results of multiple linear regression and Spearman correlation analysis showed that individual-level SES was a potential factor that may significantly affect social distance from PWMI, though the variance and correlation coefficient were relatively small. Individuals with higher SES were more likely to have lower social distance from PWMI. This finding is consistent with a previous study showing that the individual-level SES, occupation, and educational level of respondents influenced their attitude towards PWMI [1]. As early as 1981, Taylor and Dear found that more positive attitudes were linked to higher SES [40]. Similar to Taylor and Dear, Leiderman et al. found that people with higher SES showed lower social distance from people with schizophrenia. One possible explanation is that higher SES entails better education, income and occupations. Generally, people with higher individual-level SES are more likely to have better mental health literacy and objective perceptions of mental illness [41], which reduces their desire for social distance.

Moreover, the study found that an individual who was a caregiver or family member of PWMI showed lower social distance from PWMI. Those with contact with PWMI likely have a better understanding of mental illness, and they may be more familiar with or have more contact with PWMI as a friend, family member or caregiver. Those caregivers or family members are more tolerant of aggressive, negative or bizarre behaviour and are less likely to report negative attitudes and social distance. This finding is consistent with studies reporting that individuals who have a friend or family member with similar problems tended to have lower social distance [34, 42].

Familiarity or contact with PWMI was significantly related to positive attitudes in a student sample [23, 43], in the Slovak population [1] and in professional samples [44]. Many studies have revealed that contact with PWMI can be an effective anti-stigma intervention [45, 46]. However, Pranckeviciene et al. found that contact with PWMI was significantly related to lower social distance for students but not for professionals [22]. This finding illustrated that the degree of intimacy and the characteristics of the contact may lead to different attitudes towards PWMI. Further research needs to focus on the level of contact, intimacy and the ways in which the characteristics of intimacy and contact affect social distance. In addition, the effects of the interactions between familiarity and SES on stigma outcomes were not significant in this study, a finding that is inconsistent with a prior study [47]. This difference may be related to the different ways of measuring familiarity, SES and stigma. Further research focusing on the effects of the interactions between familiarity and SES on stigma outcomes is needed.

This study also found that individuals over 60 years old held more negative attitudes towards PWMI. Moreover, people with technical secondary school and higher education showed more positive attitudes towards PWMI than those with a high school education or lower. This finding was consistent with previous studies [48, 49]. Interestingly, this study revealed that people with 9 or fewer years of educational attainment held more negative attitudes towards PWMI. This could be explained by China’s nine-year education system that is compulsory for all school-age children and juveniles. This system is a public welfare undertaking implemented on July 1, 2006. The study also confirmed that education was helpful for reducing social distance.

### Scores indicating social distance between the general public and PWMI

The results suggested that social distance from PWMI was greater than the social distance found for Fukuoka participants (10.53 ± 3.44) in Japan [39]. One possible explanation is the Chinese concept of ‘face’, which describes a person’s moral standing in society [50]. Having a mental illness may mean ‘losing face’, which can greatly affect one’s access to social capital and bring shame to oneself and one’s family. Chinese individuals may thus feel more threatened by mental illness as a mark of shame, which in turn leads to greater feelings of physical threat and more desire for social distance from PWMI [27]. This finding is consistent with other studies [27, 28, 39].

Another possible explanation for the differences between the present study and the Japanese study is the psychiatric facilities. Against this backdrop of psychiatric treatment and the Japanese medical insurance system, almost all schizophrenia patients either are admitted to or attend a psychiatric hospital [39]. The general public has few opportunities for contact with PWMI. By contrast, in China, because of the imbalanced distribution of the mental health workforce and facilities [51, 52] and low help-seeking rates (approximately 5%) [52, 53], there are more opportunities for the general public to meet PWMI in daily life. For untreated PWMI, the disease may affect their daily behaviours, leading to a poor prognosis. The general public may witness such incidents or hear about their occurrence in the region, which could lead to stereotypes. Thus, the social distance from PWMI may increase.

### Relevance for clinical practice

PWMI often suffer from public stigma, making them unwilling to seek help and reducing their access to early treatment. This article reveals that public stigma towards PWMI is common in Southwest China. More anti-stigma interventions are necessary for individuals who have low levels of education, individual-level SES or contact with PWMI. The findings provide healthcare workers with important information regarding the need to carry out anti-stigma campaigns targeting increasing contact with PWMI and specific groups, such as those with low education levels and low individual-level SES.

## Limitations

Several factors may have limited our study. First, this was a cross-sectional study, so it is hard to determine the relationships between the sociodemographic variables and social distance. Second, more than 30% of the participants reported being caregivers for PWMI in this study, a figure that seems high. Since the study was conducted in a single site, it cannot fully represent the entire Chinese population. We should be aware of the bias in the sample selection. Finally, the variance and correlation coefficients were quite low. All these factors limit the generalizability of the findings; however, we believe that this study adds to the body of knowledge regarding social distance from PWMI among the general public.

## Conclusion

The findings enhance the literature on public attitudes towards PWMI and highlight the importance of understanding these issues in mainland China. This study reveals that public stigma towards PWMI is common in Southeast China. Furthermore, the results suggest that reducing public stigma towards PWMI involves considering several factors, for example, contact with PWMI. Anti-stigma interventions must be provided for individuals who are not family members or caregivers of PWMI or who have low levels of education or individual-level SES.

## Supplementary Information

**Additional file 1.** The assigned values of categorical variables.

## Data Availability

The datasets used during the current study are available from the corresponding author on reasonable request.

## Abbreviations

PWMI: People with mental illness
SDS: Social Distance Scale
SDSC: The Chinese version of Social Distance Scale
SES: Socioeconomic status
ANOVA: Analysis of Variance
