# Supplementary material for: Individual-level socioeconomic status and contact or familiarity with people with mental illness: a cross-sectional study in Wuhou District, Chengdu, Southwest China
Source: BMC Fam Pract. 2021 Apr 9;22:71. doi: 10.1186/s12875-021-01422-y (PMC8035729; doi:10.1186/s12875-021-01422-y)
Supplement: Supplementary file 1 — Additional file 1. The assigned values of categorical variables. [file 12875_2021_1422_MOESM1_ESM.pdf]

Additional file 1 The assigned values of categorical variables.

| Categorical variables | Value |
|---|---|
| Gender | |
| Male | 0 |
| Female | 1 |
| Age group (year) | |
| 18-30 | 0,0,0,0 |
| 31-45 | 1,0,0,0 |
| 46-60 | 0,1,0,0 |
| 61-75 | 0,0,1,0 |
| More than 75 | 0,0,0,1 |
| Marital status | |
| Unmarried | 0,0 |
| Married or live together | 1,0 |
| Divorced or widowed | 0,1 |
| Occupation | |
| Large company managers, public institutions, or professional and technical personnel | 1 |
| Small company managers or assistive technicians | 2 |
| Ordinary staff or clerks | 3 |
| Skilled worker | 4 |
| Farmers, temporary workers or unemployed individuals | 5 |
| Current residence | |
| Urban | 0 |
| Town or rural area | 1 |
| Educational attainment (year) | |
| Less than or equal to 9 | 0 |
| More than 9 | 1 |
| Are you a caregiver of people with mental illness? | |
| Yes | 0 |

| | |
|---|---|
| No | 1 |
| Do you have a family member who has experienced mental illness? | |
| Yes | 0 |
| No | 1 |
| Have you experienced a mental illness? | |
| Yes | 0 |
| No | 1 |
| Socioeconomic status | |
| Low class | 1 |
| Medium-low class | 2 |
| Medium class | 3 |
| Medium-high class and High class | 4 |
| Contact or familiarity with PWMI group | |
| No-contact or familiarity with PWMI at all | 1 |
| Contact or familiarity with PWMI | 2 |
| All-contact or familiarity with PWMI | 3 |
